# Early Operation for Adenoids

**Published:** 1908-05

**Authors:** Alex W. Stirling

**Affiliations:** M. D., C. M., (Edin.) D. P. H. (London). Oculist and aurist to the Wesley Memorial, Presbyterian and Tabernacle Hospitals, and to the Atlanta, Birmingham and Atlanta Railway Company, Atlanta, Ga.


### EARLY OPERATION FOR ADENOIDS.*

BY ALEX W. STIRLING, M. D., C. M., (Edin.) D. P. H. (London).
OCULIST AND AURIST TO THE WESLEY MEMORIAL, PRESBYTERIAN AND TABERNACLE HOSPITALS, AND TO THE ATLANTA, BIRMINGHAM AND ATLANTIC RAILWAY COMPANY,
ATLANTA, GA.

It may possibly appear to some that one who in these days ventures to write even a short paper upon the subject of adenoids thereby makes himself a public nuisance, and should at least apologize for his temerity.

It is to be hoped, therefore, that the subject matter of this communication may prove its own sufficient excuse. For in spite of all that has been said of nasal obstruction, I believe that, in relation to the matter upon which I propose to dwell for a few moments, even now its dire effects have not been thoroughly appreciated. I shall pass lightly over the digestive symptoms produced by the swallowing of post-nasal secretions; the fever; the disturbed sleep, due to insufficient aeration of the blood; and the natural attempt to breathe with the mouth shut; the consequent listlessness and aprosexia; the enuresis, explicable on the theory that the nerve centres are dulled as by nitrous oxide, or, perhaps reflex, like the not uncommon hay fever, asthma, and stammering; the enlarged glands behind and perhaps in front of the sternomastoid muscle; the enhanced liability to and danger from such diseases as scarlet fever, diphtheria, bronchitis, etc.;the frequent deterioration of the general health, as shown by anemia, stunted growth, headaches, night sweats, and so on, brought about by a combination of the various ingredients in the pathology of the whole condition, a vicious circle having been set up; the nasal "catarrh" along with the Eustachian infection, middle ear inflammation, deafness, and perhaps mastoid involvement, which we know to be common incidents in the lives of adenoid subjects.

*Read before Georgia State Medical Association, April, 1908

This cycle of events is already fairly familiar to all practitioners of medicine, and even the laity is beginning to look upon "asteroids," "aneroids," or whatever may be the name by which they know the offending growth, as one of the numerous penalties which afflict such as uphold the nation by multiplying the family.

But there is one feature of the mouth breathing question which has not yet become nearly so indelibly impressed upon either the medical or the lay mind as it deserves. I refer to the permanent deformities which follow upon it, if it exist while the facial or thoracic bones are still in the plastic stage. These deformities have been, by certain French authorities, laid at the door of rickets, and even syphilis. The latter is certainly a very rare cause, but it may be that there is some relationship between the osseous changes and rickets, while rachitic bones are doubtless especially liable to alteration in shape from external pressure.

In discussing these changes, it may first be stated in general terms that the mouth is related to the digestive, the nose to the respiratory system, and it ought to be looked upon as nearly as outrageous to breathe through the mouth as it would be to drink through the nose. Circumstances make the former, however, an all too common proceeding, and with disastrous, if insidious effects, except when used as an assistance to normal nasal breathing during extraordinary physical effort. As regards the facial deformity, the *crux* of the affair lies with the palate. The palate is moulded into shape mainly by two forces: It is arched by the lateral compression of the buccal muscles; and its arch is broadened and shallowed by the action of the tongue below. At the period of the second dentition, round about the seventh year, and especially in the case of the long-faced types of humanity, the jaws are undergoing a rapid development with a new eruption of teeth in view. Then it is of importance that nothing should interfere with that development. But any form of nasal obstruction will, and adenoids, the result of previous nasal inflammations, constitute the most potent factor in the causation of the pathological events mentioned, though not the only one.

As the air cannot pass freely through the nose, in spite of the instinctive earnest endeavors of the child, the mouth must of necessity be left open. The lateral compression of the facial muscles is thus increased by the strain of the hanging jaw; and not only that, but the tongue is removed from contact with the palate, upon which in normal circumstances it keeps up a steady modifying pressure outwards, a pressure which is probably more powerful than is generally appreciated.

When the period arrives for the eruption of the second dentition the young teeth must make the best of circumstances, and accordingly instead of appearing in regular contour along the margins of the jaw, they push themselves out in the line of least resistance, each fighting its neighbors for a place, till they may look as if they had been sown broadcast over the aveoli. The smile of affection is likely to be modified by the full disclosure of such an unattractive mouth, and the marital prospects of the owner are accordingly diminished. Besides which, the teeth themselves cannot be completely cleaned, and are prone to early decay.

But the evil programme does not end here. The nose is probably also seriously affected, and for a reason which is easily demonstrable. It is like the eye, a double organ, the division being effected through the interpolation of a series of cartilages and bones. These take no note of the pathological process going on in the mouth, and like the teeth, insist upon appearing in their full number and dimensions. But the place to which they have a natural right has been shrunken by the upward arching of the hard palate, and they, too, must perforce squeeze themselves in the best way they can, and that is often a very unpleasant way for their owner. They must be content to undergo contortions which appear to the examining eye as the various forms of deviation, many of which require resection if the nose to which they belong is ever adequately to perform its duty. Thus is set up another segment in the vicious circle which leads to deformity—mouth breathing reacting on itself by continually enhancing its necessity, and making more and more unavoidable operative interference in order that the cycle may be broken. Atrophy of the muscles of the nostril, which should act mildly after the manner of those of the horse, result in collapse and dimpling of the *alae nasi,* and this, combined with the immobile upper and the too prominent lower lip and the open mouth, go to make up what has been called the "adenoid facies."

But the face is not the only region deformed by the action of nasal obstruction. The chest may also suffer in a manner dependent upon the age of the sufferer. After the osseous tissue becomes well established in the ribs and sternum, the effort to breathe normally through the mouth results merely in a flattening of the chest walls, and an indrawing of the inferior portion of the sternum. But in earlier years the tendency is to the formation of "Harrison's (transverse) furrow," and to the depression of the costal cartilages, with prominence of the sternum, called "Pigeon-breast," which is likely to be more marked the more vigorous the effort to breathe through the nose. And this difficulty of breathing is increased by enlargement of the faucial tonsils. The *modus operandi* of the deformity is apparent enough. A child will always do his utmost to breathe through his nose, even when his mouth is open, before the mouth breathing habit has been finally established. The result is often stridor, sometimes mistaken for croup, and, of course, an indrawing of the delicate walls of the chest, which in time becomes permanent.

The effects of mouth breathing, I think then you will agree with me, are not only powerful to destroy the healthy contour of the face and chest, but may also bring in their wake such danger and suffering as indicate early operation for their prevention.

